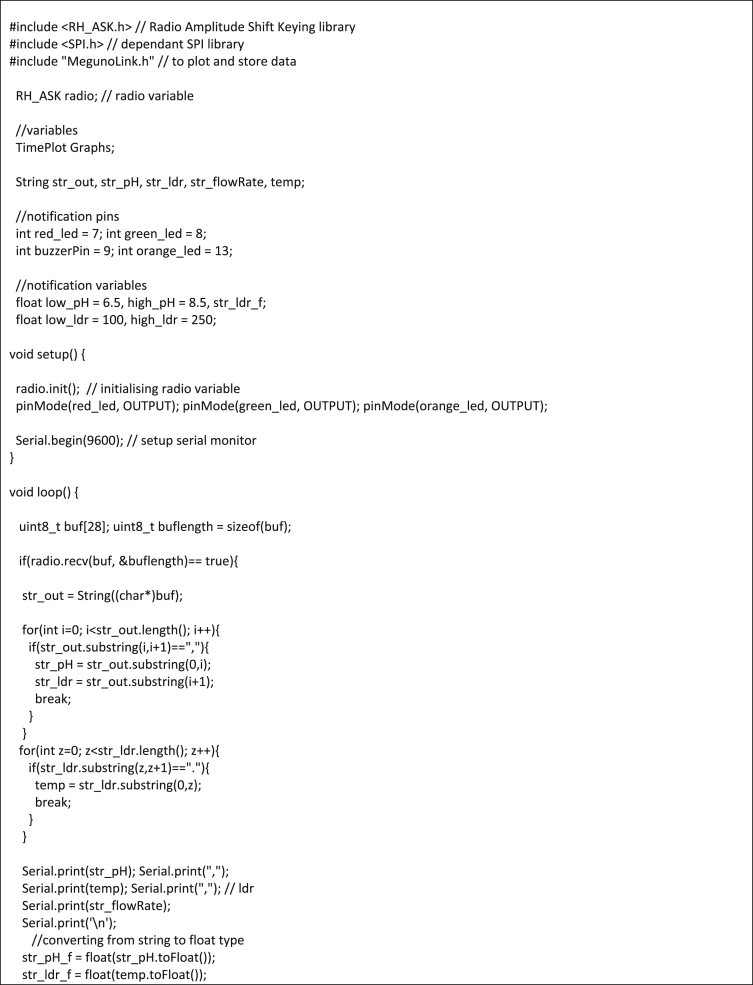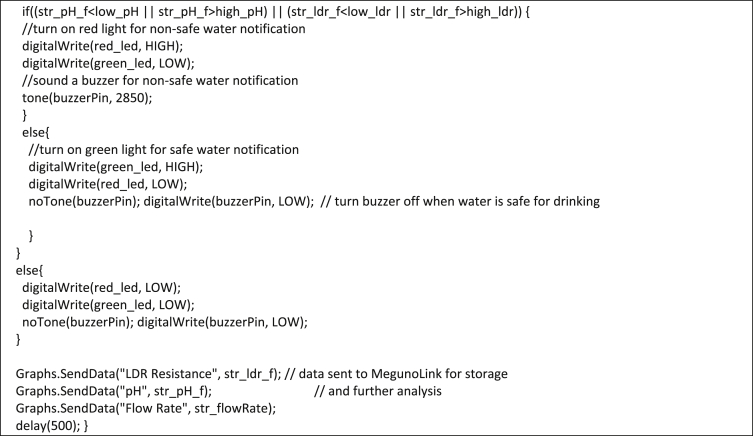# Dataset for a wireless sensor network based drinking-water quality monitoring and notification system

**DOI:** 10.1016/j.dib.2019.104813

**Published:** 2019-11-16

**Authors:** Mhambi Phila Philadephian Sithole, Nnamdi I. Nwulu, Eustace M. Dogo

**Affiliations:** Department of Electrical and Electronic Engineering Science, University of Johannesburg, South Africa

**Keywords:** Water quality parameters, Sensors, Internet of things, Wireless communication, Micro-controller, Water monitoring, Water safety

## Abstract

This paper presents the collected experimental data for water quality monitoring which was conducted in ten experiments by using five different common sources of water contaminants namely soil, salt, washing powder, chlorine and vinegar and their combination. The data were collected indoors at room temperature during the day for several days using sensors that measure pH, turbidity, flow rate, and conductivity in water. The water consumption risk (CR) was calculated as deviation based on the water quality parameters standards proposed by the World Health Organisation (WHO) and the South African Department of Water Affairs (DWA), with respect to the sensor measurement readings obtained. While the error measurements were calculated based on the expected parameter measurement per conducted experiment and repeated for 26 measurements. Pure tap water was the benchmark of water safe for human consumption. The first five experiments were performed by introducing each contaminant into the water and thereafter, two contaminants in the sixth experiment and their additions until all different contaminants were experimented at once in the last experiment.

**Table:** Specifications Table

Subject	Computer Networks and Communications, Engineering
Specific subject area	Application of computing network and Engineering in monitoring water quality safety, risk and reliability.
Type of data	Tables and Graphs
How data were acquired	Data was captured using sensors and sent wirelessly with an HFY-FST radio transmitter module, then it was received with an HFY-J18 radio receiver module and analysed (graphically and tabularly) through MegunoLink interface tool. Both the transmitter and receiver were implemented with identical Arduino Uno R3 microcontroller boards.
Data format	Raw and analysed sensors data
Parameters for data collection	Room temperature at 25 °C, with constant illumination room (constant light). The data was collected during the day.
Data source location	University of Johannesburg, Auckland Park Campus, Johannesburg, South Africa. Latitude and longitude (and GPS coordinates): S26 10 54.9 E27 59 53.9
Data accessibility	Public data repository: Dataset is available at Mendeley data. Data Digital Object Identifier number: https://doi.org/10.17632/pbb76sbbrg.1 Direct URL to data: https://data.mendeley.com/datasets/pbb76sbbrg/1 Dataset citation: Sithole, Mhambi Phila Philadephian; Nwulu, Nnamdi; Dogo, Eustace (2019), “Wireless Sensor Network Based Drinking-Water Quality Monitoring and Notification System Dataset”, Mendeley Data, v1 https://doi.org/10.17632/pbb76sbbrg.1. Also, in Ref. [1].
Related research article	The data article is related to this [2] research article

**Table:** 

**Value of the Data** • The dataset presented in this paper can be used for further experiments on water quality monitoring as leverage using data mining methods such as machine learning [3] and emerging technologies such as IoT and blockchain for water quality monitoring and management [4]. • It can also be used to validate experimental data of the same nature as it is of significant quality and it was validated using international standards. • Another value of this data is in water purification, as it contains ratios of contaminants which are very useful in water purification and can be used in the water industry.

## 1 Data

**Fig. 1:**
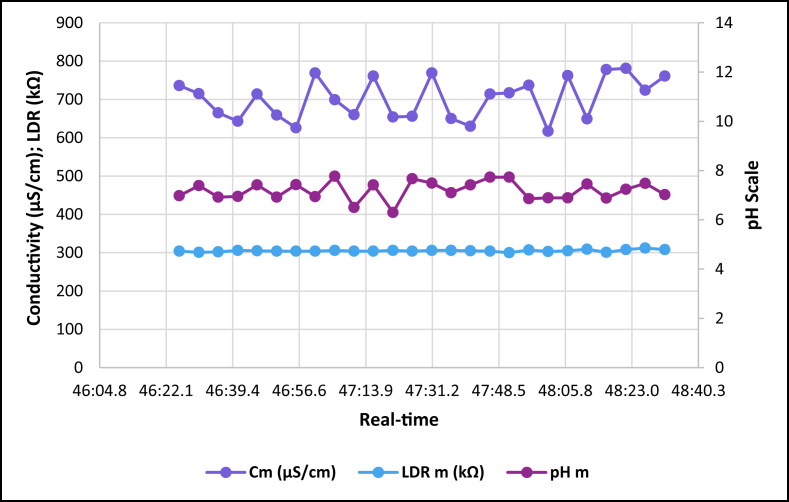
Pure tap water quality parameters.

**Fig. 2:**
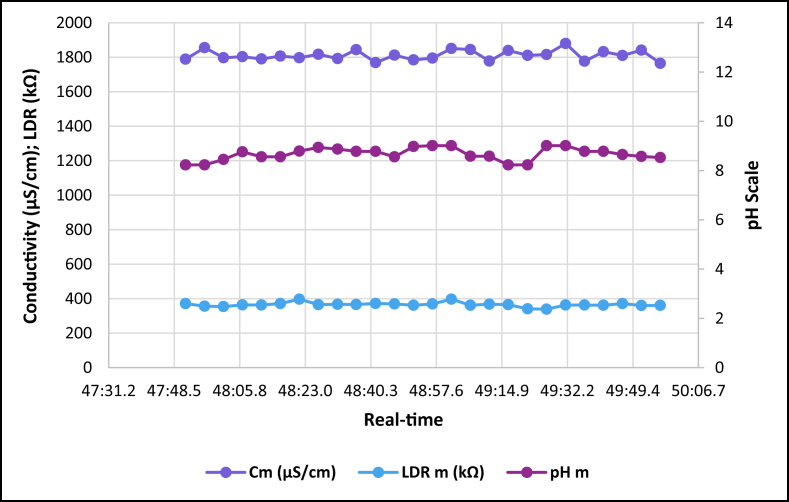
Salt contaminated tap water parameters.

**Fig. 3:**
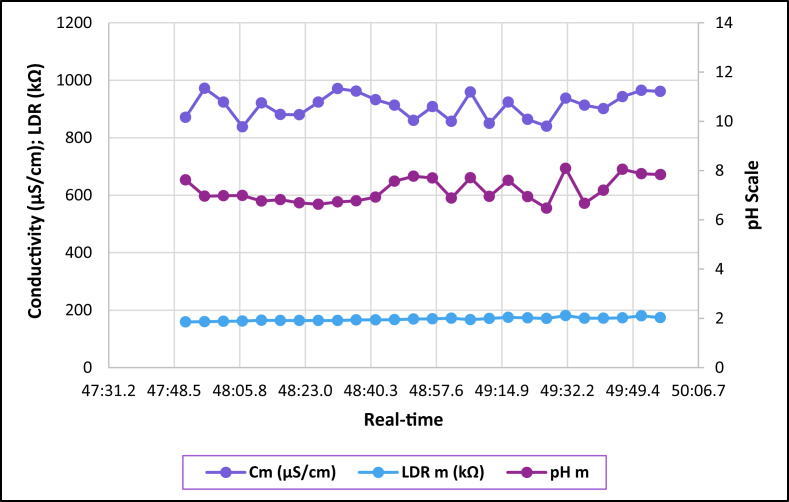
Soil contaminated tap water parameters.

**Fig. 4:**
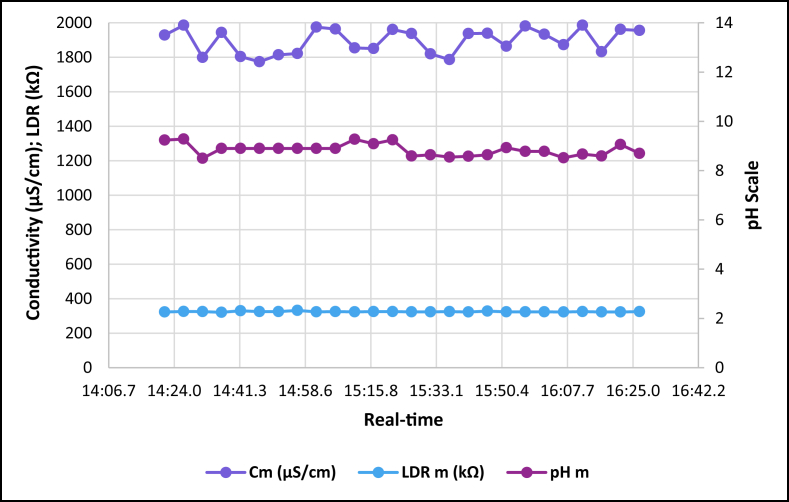
Chlorine contaminated tap water parameters.

**Fig. 5:**
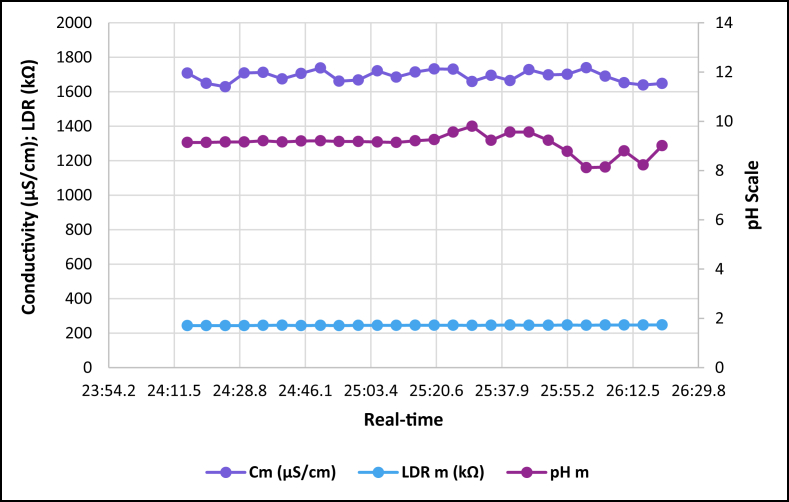
Washing powder contaminated tap water parameters.

**Fig. 6:**
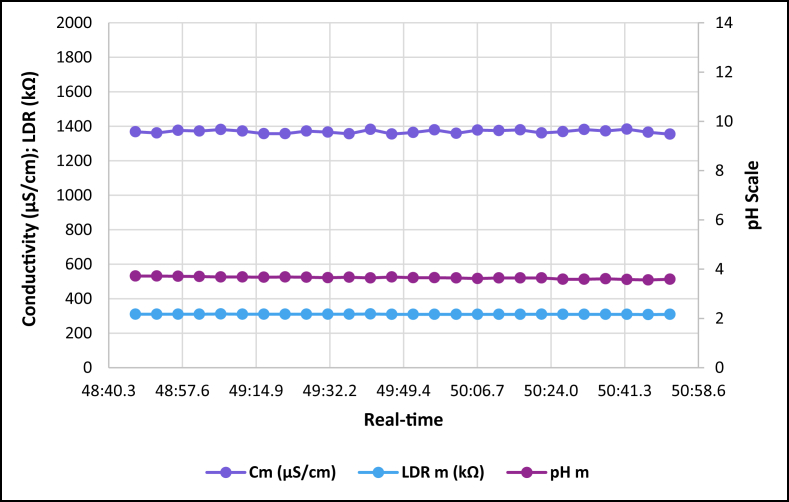
Vinegar contaminated tap water parameters.

**Fig. 7:**
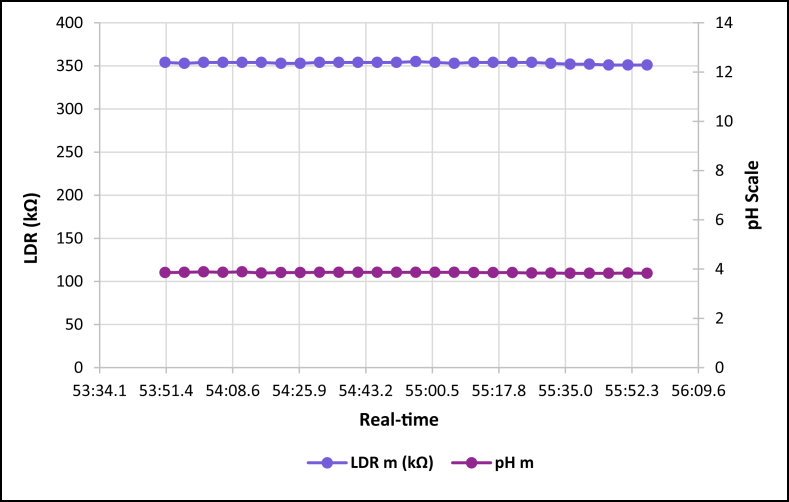
Vinegar + washing powder contaminated tap water parameters.

**Fig. 8:**
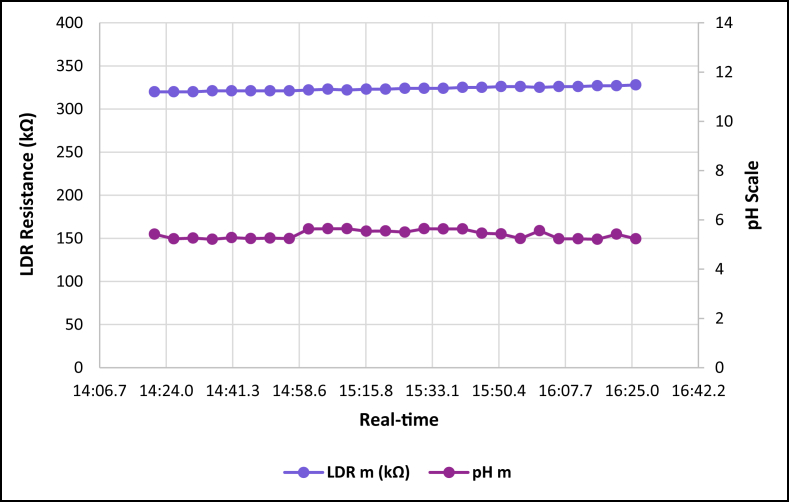
Vinegar + washing powder + chlorine contaminated tap water parameters.

**Fig. 9:**
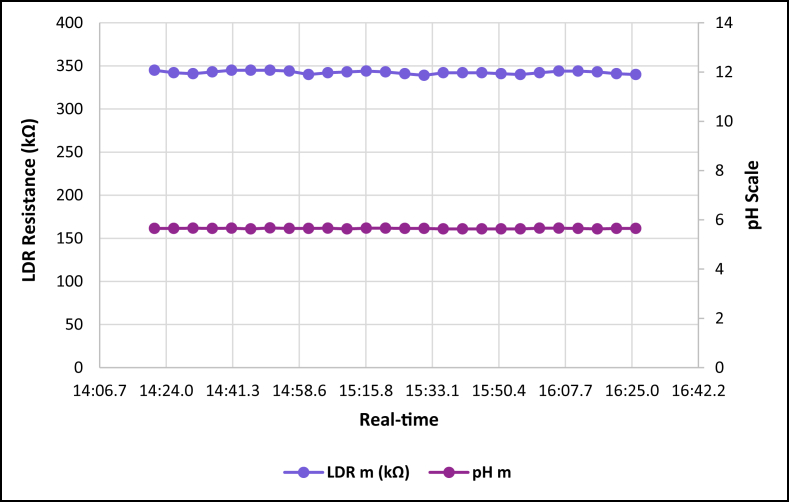
Vinegar + washing powder + chlorine + salt contaminated tap water parameters.

**Fig. 10:**
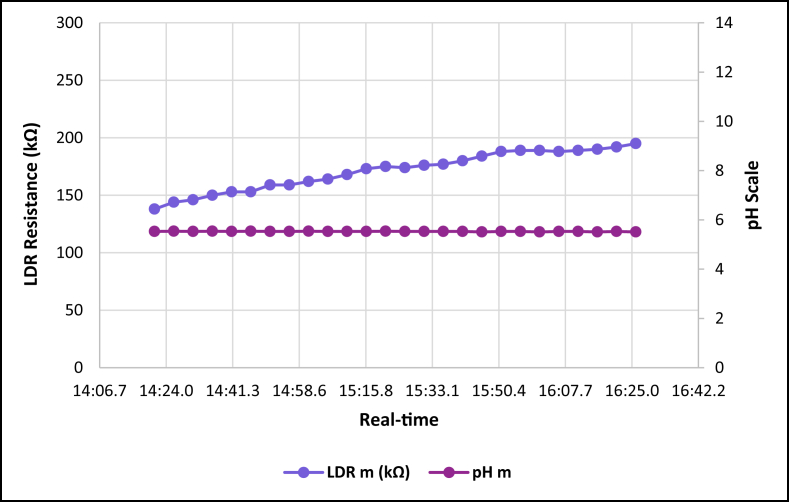
Vinegar + washing powder + chlorine + salt + soil contaminated tap water parameters.

The dataset is published online in the Mendeley data repository [1]. The presented data were collected for all ten experiments conducted with the first being data for pure tap water and the rest being data for contaminated water using different contaminants and their additions until all different contaminants were experimented at once. Table 1 present the benchmark WHO standards of water parameters for safe human consumable water. The graphs portray the trends of each experiment showing the change in parameter values due to introduction of contaminant(s) with scaling for pH, conductivity and LDR (Light Dependant Resistor) and real-time scaling as data was collected in real-time. The real-time measurement values are presented in Table 2, Table 3, Table 4, Table 5, Table 6, Table 7, Table 8, Table 9, Table 10, Table 11. Conductivity, pH and LDR resistance (representing turbidity) were measured for the first six experiments and only pH and LDR values were measured for the last four experiments because the values were beyond the conductivity meter rating of 0–1999 μS/cm. Fig. 1 portrays the trends for pure tap water and their range, which is the benchmark quality parameters used in comparison to WHO water quality standards. Furthermore, it is vital that the water quality ranges of the water used must be analysed and known in order to ensure the quality and validity of the results. Fig. 2 shows the trends for soil contaminated water. Fig. 3 shows the trends for chlorine contaminated water. Fig. 4 depicts salt contaminated water parameter trends. Fig. 5 shows washing powder contaminated water trends. Fig. 6 shows vinegar contaminated water trends. Trends for vinegar + washing powder contaminated water are portrayed in Fig. 7. Fig. 8 shows the trends for vinegar + washing powder + chlorine contaminated water. Trends for vinegar + washing powder + chlorine + salt contaminated water are presented in Fig. 9. Fig. 10 shows the trends for vinegar + washing powder + chlorine + salt + soil contaminated water.

**Table 1:** Water quality parameters proposed by WHO and South Africa, DWA [5,6].

S/N	Parameter	Quality Range	Units
1	pH	6.5–8.5	pH
2	Electrical Conductivity	500–1000	μS/cm
3	Turbidity	0–5	NTU
4	ORP	650–800	mV
5	Temperature	–	^o^C
6	Free Residual Chlorine	0.2–2	mg/L
7	Dissolved Oxygen	–	mg/L
8	Nitrates	<10	mg/L

**Table 2:** Pure tap water real-time readings and analysis.

Real-Time	pH m	pH CR	Cm (μS/cm)	Conductivity CR	LDR m (kΩ)	LDR CR
46:25.5	6.98	No Risk	736	No Risk	304	No Risk
46:30.6	7.39	No Risk	715	No Risk	301	No Risk
46:35.6	6.92	No Risk	665	No Risk	302	No Risk
46:40.7	6.95	No Risk	643	No Risk	306	No Risk
46:45.7	7.42	No Risk	714	No Risk	305	No Risk
46:50.8	6.92	No Risk	659	No Risk	304	No Risk
46:55.8	7.43	No Risk	626	No Risk	304	No Risk
47:00.8	6.94	No Risk	769	No Risk	304	No Risk
47:05.9	7.77	No Risk	699	No Risk	306	No Risk
47:10.9	6.5	No Risk	660	No Risk	304	No Risk
47:15.9	7.42	No Risk	761	No Risk	304	No Risk
47:21.0	6.3	1.43%	654	No Risk	306	No Risk
47:26.0	7.67	No Risk	656	No Risk	304	No Risk
47:31.1	7.49	No Risk	769	No Risk	306	No Risk
47:36.1	7.1	No Risk	650	No Risk	306	No Risk
47:41.1	7.42	No Risk	630	No Risk	305	No Risk
47:46.2	7.73	No Risk	714	No Risk	304	No Risk
47:51.2	7.73	No Risk	717	No Risk	300	No Risk
47:56.3	6.86	No Risk	737	No Risk	307	No Risk
48:01.3	6.89	No Risk	617	No Risk	303	No Risk
48:06.4	6.89	No Risk	762	No Risk	305	No Risk
48:11.4	7.45	No Risk	649	No Risk	309	No Risk
48:16.4	6.88	No Risk	778	No Risk	301	No Risk
48:21.5	7.24	No Risk	781	No Risk	308	No Risk
48:26.5	7.48	No Risk	724	No Risk	312	No Risk
48:31.6	7.02	No Risk	761	No Risk	308	No Risk

Analysis				
Total measurements	26	26	Min LDR	300
Fault measurements	1	0	Max LDR	312
Error in measurements (%)	3.85%	0.00%		
Total CR	1.43%	0.00%

**Table 3:** Soil contaminated water real-time readings and analysis.

Real-Time	pH m	pH CR	Cm (μS/cm)	Conductivity CR	LDR m (kΩ)	LDR CR
47:51.5	7.62	No Risk	871	No Risk	159	28.20%
47:56.5	6.96	No Risk	972	No Risk	160	28.00%
48:01.5	6.98	No Risk	924	No Risk	161	27.80%
48:06.5	6.99	No Risk	838	No Risk	162	27.60%
48:11.5	6.76	No Risk	921	No Risk	165	27.00%
48:16.5	6.82	No Risk	881	No Risk	164	27.20%
48:21.5	6.69	No Risk	880	No Risk	164	27.20%
48:26.5	6.63	No Risk	924	No Risk	164	27.20%
48:31.6	6.73	No Risk	971	No Risk	164	27.20%
48:36.5	6.77	No Risk	962	No Risk	166	26.80%
48:41.6	6.92	No Risk	932	No Risk	166	26.80%
48:46.6	7.57	No Risk	913	No Risk	167	26.60%
48:51.6	7.77	No Risk	860	No Risk	169	26.20%
48:56.6	7.7	No Risk	908	No Risk	170	26.00%
49:01.6	6.88	No Risk	857	No Risk	172	25.60%
49:06.6	7.71	No Risk	959	No Risk	167	26.60%
49:11.6	6.95	No Risk	850	No Risk	171	25.80%
49:16.6	7.6	No Risk	924	No Risk	175	25.00%
49:21.7	6.94	No Risk	864	No Risk	173	25.40%
49:26.7	6.47	0.20%	840	No Risk	171	25.80%
49:31.7	8.09	No Risk	938	No Risk	181	23.80%
49:36.7	6.67	No Risk	913	No Risk	172	25.60%
49:41.7	7.2	No Risk	901	No Risk	172	25.60%
49:46.7	8.05	No Risk	943	No Risk	173	25.40%
49:51.7	7.87	No Risk	965	No Risk	180	24.00%
49:56.7	7.84	No Risk	961	No Risk	174	25.20%

Analysis				
Total measurements	26	25	169	26
Fault measurements	1	0		0
Error in measurements (%)	3.85%	0.00%		0.00%
Total CR	0.20%	0.00%		26.29%

**Table 4:** Chlorine contaminated water real-time readings and analysis.

Real-Time	pH m	pH CR	Cm (μS/cm)	Conductivity CR	LDR m (kΩ)	LDR CR
14:21.5	9.24	4.93%	1929	46.45%	323	2.20%
14:26.5	9.28	5.20%	1986	49.30%	326	2.80%
14:31.5	8.5	No Risk	1799	39.95%	325	2.60%
14:36.5	8.9	2.67%	1944	47.20%	321	1.80%
14:41.5	8.9	2.67%	1804	40.20%	330	3.60%
14:46.5	8.9	2.67%	1774	38.70%	325	2.60%
14:51.5	8.9	2.67%	1814	40.70%	325	2.60%
14:56.5	8.9	2.67%	1822	41.10%	332	4.00%
15:01.5	8.9	2.67%	1975	48.75%	324	2.40%
15:06.5	8.9	2.67%	1964	48.20%	325	2.60%
15:11.6	9.27	5.13%	1854	42.70%	324	2.40%
15:16.6	9.09	3.93%	1851	42.55%	325	2.60%
15:21.6	9.25	5.00%	1961	48.05%	325	2.60%
15:26.6	8.59	0.60%	1938	46.90%	324	2.40%
15:31.6	8.64	0.93%	1820	41.00%	324	2.40%
15:36.6	8.55	0.33%	1787	39.35%	325	2.60%
15:41.6	8.58	0.53%	1938	46.90%	323	2.20%
15:46.6	8.64	0.93%	1939	46.95%	328	3.20%
15:51.6	8.93	2.87%	1864	43.20%	324	2.40%
15:56.6	8.78	1.87%	1981	49.05%	324	2.40%
16:01.6	8.78	1.87%	1934	46.70%	324	2.40%
16:06.7	8.52	0.13%	1873	43.65%	323	2.20%
16:11.7	8.67	1.13%	1986	49.30%	325	2.60%
16:16.7	8.59	0.60%	1832	41.60%	323	2.20%
16:21.7	9.06	3.73%	1962	48.10%	323	2.20%
16:26.7	8.7	1.33%	1956	47.80%	325	2.60%

Analysis				
Total measurements	26	26	325	26
Fault measurements	1	0		0
Error in measurements (%)	3.85%	0.00%		0.00%
Total CR	2.39%	44.78%		2.56%

**Table 5:** Salt contaminated water real-time readings and analysis.

Real-Time	pH m	pH CR	Cm (μS/cm)	Conductivity CR	LDR m (kΩ)	LDR CR
47:51.5	8.23	No Risk	1789	39.45%	371	11.80%
47:56.5	8.23	No Risk	1856	42.80%	356	8.80%
48:01.5	8.45	No Risk	1797	39.85%	354	8.40%
48:06.5	8.76	1.73%	1803	40.15%	364	10.40%
48:11.5	8.56	0.40%	1790	39.50%	363	10.20%
48:16.5	8.56	0.40%	1807	40.35%	371	11.80%
48:21.5	8.79	1.93%	1797	39.85%	397	17.00%
48:26.5	8.94	2.93%	1817	40.85%	365	10.60%
48:31.6	8.87	2.47%	1792	39.60%	367	11.00%
48:36.5	8.78	1.87%	1844	42.20%	366	10.80%
48:41.6	8.78	1.87%	1769	38.45%	372	12.00%
48:46.6	8.56	0.40%	1812	40.60%	369	11.40%
48:51.6	8.98	3.20%	1785	39.25%	362	10.00%
48:56.6	9.01	3.40%	1795	39.75%	369	11.40%
49:01.6	9.01	3.40%	1851	42.55%	397	17.00%
49:06.6	8.58	0.53%	1845	42.25%	362	10.00%
49:11.6	8.58	0.53%	1778	38.90%	368	11.20%
49:16.6	8.23	No Risk	1839	41.95%	365	10.60%
49:21.7	8.23	No Risk	1811	40.55%	341	5.80%
49:26.7	9.01	3.40%	1816	40.80%	339	5.40%
49:31.7	9.01	3.40%	1880	44.00%	363	10.20%
49:36.7	8.78	1.87%	1777	38.85%	363	10.20%
49:41.7	8.78	1.87%	1832	41.60%	362	10.00%
49:46.7	8.65	1.00%	1810	40.50%	371	11.80%
49:51.7	8.57	0.47%	1841	42.05%	360	9.60%
49:56.7	8.53	0.20%	1765	38.25%	361	9.80%

Analysis				
Total measurements	26	25	365	26
Fault measurements	5	0		0
Error in measurements (%)	19.23%	0.00%		0.00%
Total CR	1.77%	40.57%		10.66%

**Table 6:** Washing powder contaminated real-time readings and analysis.

Real-Time	pH m	pH CR	Cm (μS/cm)	Conductivity CR	LDR m (kΩ)	LDR CR
24:15.0	9.14	4.27%	1708	35.40%	244	11.20%
24:20.0	9.14	4.27%	1649	32.45%	244	11.20%
24:25.0	9.16	4.40%	1629	31.45%	244	11.20%
24:30.0	9.16	4.40%	1709	35.45%	244	11.20%
24:35.0	9.21	4.73%	1712	35.60%	245	11.00%
24:40.0	9.16	4.40%	1674	33.70%	246	10.80%
24:45.0	9.2	4.67%	1706	35.30%	244	11.20%
24:50.1	9.21	4.73%	1738	36.90%	245	11.00%
24:55.0	9.18	4.53%	1661	33.05%	244	11.20%
25:00.1	9.18	4.53%	1668	33.40%	245	11.00%
25:05.1	9.16	4.40%	1721	36.05%	245	11.00%
25:10.1	9.14	4.27%	1685	34.25%	245	11.00%
25:15.1	9.21	4.73%	1714	35.70%	246	10.80%
25:20.1	9.26	5.07%	1732	36.60%	246	10.80%
25:25.1	9.56	7.07%	1731	36.55%	246	10.80%
25:30.1	9.8	8.67%	1659	32.95%	245	11.00%
25:35.1	9.23	4.87%	1695	34.75%	246	10.80%
25:40.1	9.56	7.07%	1665	33.25%	247	10.60%
25:45.1	9.56	7.07%	1728	36.40%	246	10.80%
25:50.2	9.23	4.87%	1698	34.90%	246	10.80%
25:55.2	8.78	1.87%	1701	35.05%	247	10.60%
26:00.2	8.12	No Risk	1739	36.95%	246	10.80%
26:05.2	8.14	No Risk	1690	34.50%	247	10.60%
26:10.2	8.8	2.00%	1652	32.60%	247	10.60%
26:15.2	8.23	No Risk	1639	31.95%	247	10.60%
26:20.2	9.01	3.40%	1648	32.40%	248	10.40%

Analysis				
Total measurements	26	25	246	26
Fault measurements	3	0		0
Error in measurements (%)	11.54%	0.00%		0.00%
Total CR	4.79%	34.52%		10.88%

**Table 7:** Vinegar contaminated water real-time readings and analysis.

Real-Time	pH m	pH CR	Cm (μS/cm)	Conductivity CR	LDR m (kΩ)	LDR CR
48:46.6	3.72	18.53%	1368	18.40%	310	No Risk
48:51.6	3.72	18.53%	1361	18.05%	310	No Risk
48:56.6	3.71	18.60%	1376	18.80%	310	No Risk
49:01.6	3.7	18.67%	1372	18.60%	310	No Risk
49:06.6	3.68	18.80%	1381	19.05%	311	No Risk
49:11.7	3.68	18.80%	1372	18.60%	310	No Risk
49:16.7	3.67	18.87%	1357	17.85%	310	No Risk
49:21.7	3.68	18.80%	1357	17.85%	310	No Risk
49:26.7	3.67	18.87%	1372	18.60%	310	No Risk
49:31.7	3.65	19.00%	1366	18.30%	310	No Risk
49:36.7	3.67	18.87%	1356	17.80%	310	No Risk
49:41.7	3.64	19.07%	1382	19.10%	311	No Risk
49:46.7	3.68	18.80%	1355	17.75%	309	No Risk
49:51.7	3.65	19.00%	1364	18.20%	309	No Risk
49:56.7	3.65	19.00%	1379	18.95%	309	No Risk
50:01.8	3.64	19.07%	1359	17.95%	309	No Risk
50:06.8	3.62	19.20%	1378	18.90%	309	No Risk
50:11.8	3.64	19.07%	1375	18.75%	309	No Risk
50:16.8	3.64	19.07%	1379	18.95%	309	No Risk
50:21.8	3.64	19.07%	1361	18.05%	310	No Risk
50:26.8	3.59	19.40%	1369	18.45%	309	No Risk
50:31.8	3.59	19.40%	1381	19.05%	309	No Risk
50:36.8	3.61	19.27%	1373	18.65%	309	No Risk
50:41.8	3.58	19.47%	1383	19.15%	309	No Risk
50:46.8	3.56	19.60%	1365	18.25%	308	No Risk
50:51.9	3.59	19.40%	1354	17.70%	309	No Risk

Analysis				
Total measurements	26	26	310	26
Fault measurements	0	0		0
Error in measurements (%)	0.00%	0.00%		0.00%
Total CR	19.01%	18.45%		0.00%

**Table 8:** Vinegar + washing powder contaminated water real-time readings and analysis.

Real-Time	pH m	pH CR	LDR m (kΩ)	LDR CR
53:51.1	3.86	17.60%	354	8.40%
53:56.1	3.87	17.53%	353	8.20%
54:01.1	3.89	17.40%	354	8.40%
54:06.1	3.87	17.53%	354	8.40%
54:11.1	3.89	17.40%	354	8.40%
54:16.1	3.84	17.73%	354	8.40%
54:21.2	3.86	17.60%	353	8.20%
54:26.2	3.86	17.60%	353	8.20%
54:31.2	3.87	17.53%	354	8.40%
54:36.2	3.87	17.53%	354	8.40%
54:41.2	3.87	17.53%	354	8.40%
54:46.2	3.87	17.53%	354	8.40%
54:51.2	3.87	17.53%	354	8.40%
54:56.2	3.87	17.53%	355	8.60%
55:01.2	3.87	17.53%	354	8.40%
55:06.2	3.87	17.53%	353	8.20%
55:11.3	3.86	17.60%	354	8.40%
55:16.3	3.86	17.60%	354	8.40%
55:21.3	3.86	17.60%	354	8.40%
55:26.3	3.84	17.73%	354	8.40%
55:31.3	3.84	17.73%	353	8.20%
55:36.3	3.83	17.80%	352	8.00%
55:41.3	3.83	17.80%	352	8.00%
55:46.3	3.83	17.80%	351	7.80%
55:51.3	3.84	17.73%	351	7.80%
55:56.3	3.83	17.80%	351	7.80%

Analysis			
Total measurements	26	353	26
Fault measurements	0		0
Error in measurements (%)	0.00%		0.00%
Total CR	17.61%		8.27%

**Table 9:** Vinegar + Washing powder + Chlorine contaminated water real-time readings and analysis.

Real-Time	pH m	pH CR	LDR m (kΩ)	LDR CR
14:21.0	5.42	7.20%	320	1.60%
14:26.0	5.23	8.47%	320	1.60%
14:31.0	5.26	8.27%	320	1.60%
14:36.0	5.21	8.60%	321	1.80%
14:41.0	5.28	8.13%	321	1.80%
14:46.0	5.24	8.40%	321	1.80%
14:51.0	5.26	8.27%	321	1.80%
14:56.0	5.24	8.40%	321	1.80%
15:01.0	5.63	5.80%	322	2.00%
15:06.0	5.64	5.73%	323	2.20%
15:11.0	5.64	5.73%	322	2.00%
15:16.0	5.54	6.40%	323	2.20%
15:21.0	5.55	6.33%	323	2.20%
15:26.0	5.5	6.67%	324	2.40%
15:31.0	5.64	5.73%	324	2.40%
15:36.0	5.63	5.80%	324	2.40%
15:41.0	5.63	5.80%	325	2.60%
15:46.0	5.46	6.93%	325	2.60%
15:51.0	5.43	7.13%	326	2.80%
15:56.0	5.24	8.40%	326	2.80%
16:01.0	5.56	6.27%	325	2.60%
16:06.0	5.23	8.47%	326	2.80%
16:11.0	5.23	8.47%	326	2.80%
16:16.0	5.21	8.60%	327	3.00%
16:21.0	5.42	7.20%	327	3.00%
16:26.0	5.23	8.47%	328	3.20%

Analysis			
Total measurements	26	324	26
Fault measurements	0		0
Error in measurements (%)	0.00%		0.00%
Total CR	7.29%		2.30%

**Table 10:** Vinegar + washing powder + chlorine + salt contaminated water real-time readings and analysis.

Real-Time	pH m	pH CR	LDR m (kΩ)	LDR CR
14:21.0	5.65	5.67%	345	6.60%
14:26.0	5.65	5.67%	342	6.00%
14:31.0	5.66	5.60%	341	5.80%
14:36.0	5.65	5.67%	343	6.20%
14:41.0	5.66	5.60%	345	6.60%
14:46.0	5.63	5.80%	345	6.60%
14:51.0	5.67	5.53%	345	6.60%
14:56.0	5.65	5.67%	344	6.40%
15:01.0	5.65	5.67%	340	5.60%
15:06.0	5.66	5.60%	342	6.00%
15:11.0	5.63	5.80%	343	6.20%
15:16.0	5.66	5.60%	344	6.40%
15:21.0	5.66	5.60%	343	6.20%
15:26.0	5.65	5.67%	341	5.80%
15:31.0	5.65	5.67%	339	5.40%
15:36.0	5.63	5.80%	342	6.00%
15:41.0	5.63	5.80%	342	6.00%
15:46.0	5.63	5.80%	342	6.00%
15:51.0	5.63	5.80%	341	5.80%
15:56.0	5.63	5.80%	340	5.60%
16:01.0	5.66	5.60%	342	6.00%
16:06.0	5.66	5.60%	344	6.40%
16:11.0	5.65	5.67%	344	6.40%
16:16.0	5.63	5.80%	343	6.20%
16:21.0	5.65	5.67%	341	5.80%
16:26.0	5.65	5.67%	340	5.60%

Analysis			
Total measurements	26	342	26
Fault measurements	0		0
Error in measurements (%)	0.00%		0.00%
Total CR	5.68%		6.08%

**Table 11:** Vinegar + washing powder + salt + soil contaminated water real-time readings and analysis.

Real-Time	pH m	pH CR	LDR m (kΩ)	LDR CR
14:21.0	5.53	6.47%	138	32.40%
14:26.0	5.54	6.40%	144	31.20%
14:31.0	5.53	6.47%	146	30.80%
14:36.0	5.54	6.40%	150	30.00%
14:41.0	5.53	6.47%	153	29.40%
14:46.0	5.54	6.40%	153	29.40%
14:51.0	5.53	6.47%	159	28.20%
14:56.0	5.53	6.47%	159	28.20%
15:01.0	5.54	6.40%	162	27.60%
15:06.0	5.53	6.47%	164	27.20%
15:11.0	5.53	6.47%	168	26.40%
15:16.0	5.53	6.47%	173	25.40%
15:21.0	5.54	6.40%	175	25.00%
15:26.0	5.53	6.47%	174	25.20%
15:31.0	5.53	6.47%	176	24.80%
15:36.0	5.53	6.47%	177	24.60%
15:41.0	5.53	6.47%	180	24.00%
15:46.0	5.51	6.60%	184	23.20%
15:51.0	5.53	6.47%	188	22.40%
15:56.0	5.53	6.47%	189	22.20%
16:01.0	5.51	6.60%	189	22.20%
16:06.0	5.53	6.47%	188	22.40%
16:11.0	5.53	6.47%	189	22.20%
16:16.0	5.51	6.60%	190	22.00%
16:21.0	5.53	6.47%	192	21.60%
16:26.0	5.51	6.60%	195	21.00%

Analysis			
Total measurements	26	171	26
Fault measurements	0		0
Error in measurements (%)	0.00%		0.00%
Total CR	6.47%		25.73%

## 2 Experimental design, materials, and methods

**Fig. 11:**
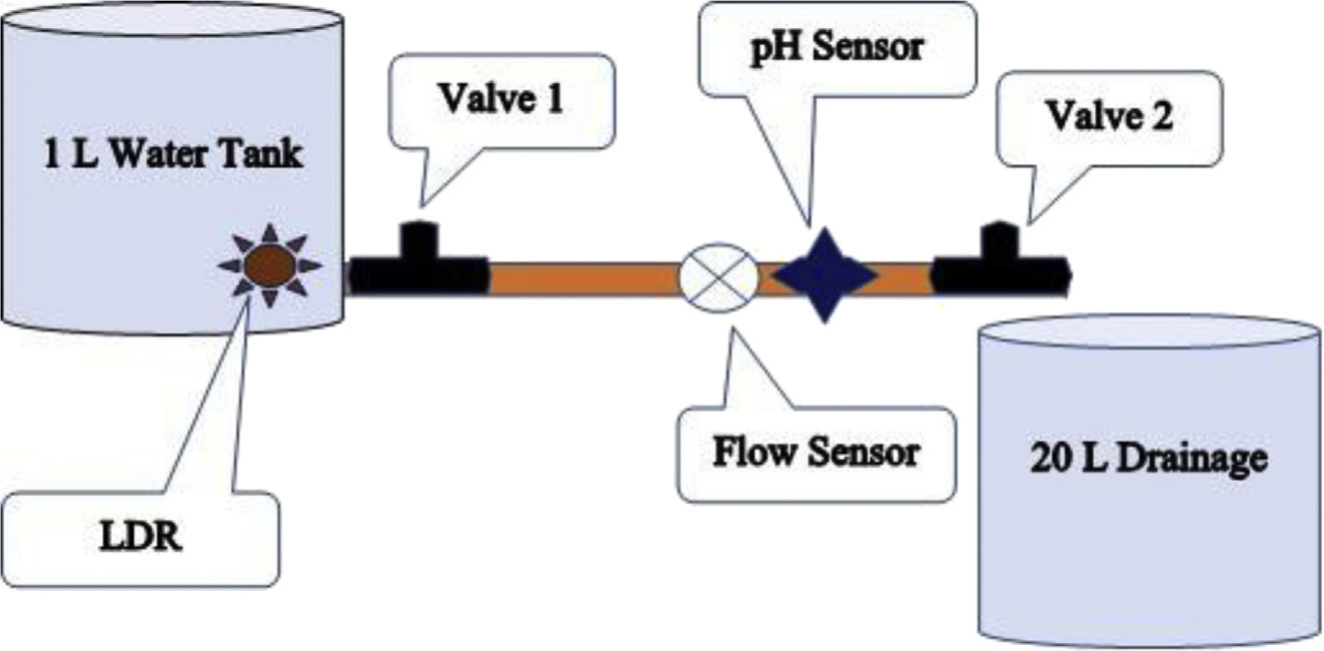
Schematic diagram of the water supply system used for data gathering.

Fig. 11 depicts the water supply design set up by which the data was gathered and analysed. It also shows how each sensor was mounted on this subsystem. All the sensors were integrated into the water supply subsystem in a way that they can accurately gather measurements from the analysed water. The pH sensor was installed inside the pipe as it functioned accurately in that location. Two valves were used to monitor and control the flow rate of water inside the pipe. The LDR was mounted on the surface of the water tank as it depends on light and since there is no light but darkness inside the pipe (where the pH sensor was mounted), otherwise the LDR would not work properly but only produce the same results for changes in water colour. The water tank was wrapped in a white paper to confine and reduce the error in measurement of the LDR for changes in water colour and for usage in indoor environment. A one (1) litre water tank was selected, to enable mobility of the system, and also to save and converse water for the duration of the testing phase. The twenty (20) litre container was used to drain both the pure tap water and the contaminated tap water after each analysis and testing.

### 2.1 List of hardware components and materials

• pH sensor
• Flow sensor
• 2× valves
• LDR
• 1L water tank
• 20L drainage bucket
• 1 m plastic pipe
• Arduino Microcontrollers
• HFY radio modules

### 2.2 Methods

Pure tap water parameters were measured first to validate the water quality standards as well as the performance of the system developed. Then five contaminants were used namely; soil, chlorine, salt, vinegar and washing powder. The soil was chosen because water can be contaminated by the soil in events of leakages on the water supply and distribution system. Chlorine was chosen because water can be overtreated and distributed without proper analysis, this is a mistake that might happen in water industries. Salt was chosen because of its ability to dissolve in water, and also to test the LDR, pH response and the conductivity. Water with high dissolved substances is not healthy for consumption, so the system must be able to detect such effects. Washing powder is known to be soapy, thus alkaline. This contaminant was chosen to test the system's response to soapy substances present in water. Vinegar is known to be a sour substance, thus acidic. It was chosen to test the system's response to acidic substances present in water. This phenomenon occurs mostly in corrosive pipes, which produces an acidic substance. Later experiments are conducted by the addition of the above-mentioned contaminants one at a time and checking the system's response for combined contaminants in water.

### 2.3 Arduino sketches (codes)

1) Measuring subsystem

**Figure:**
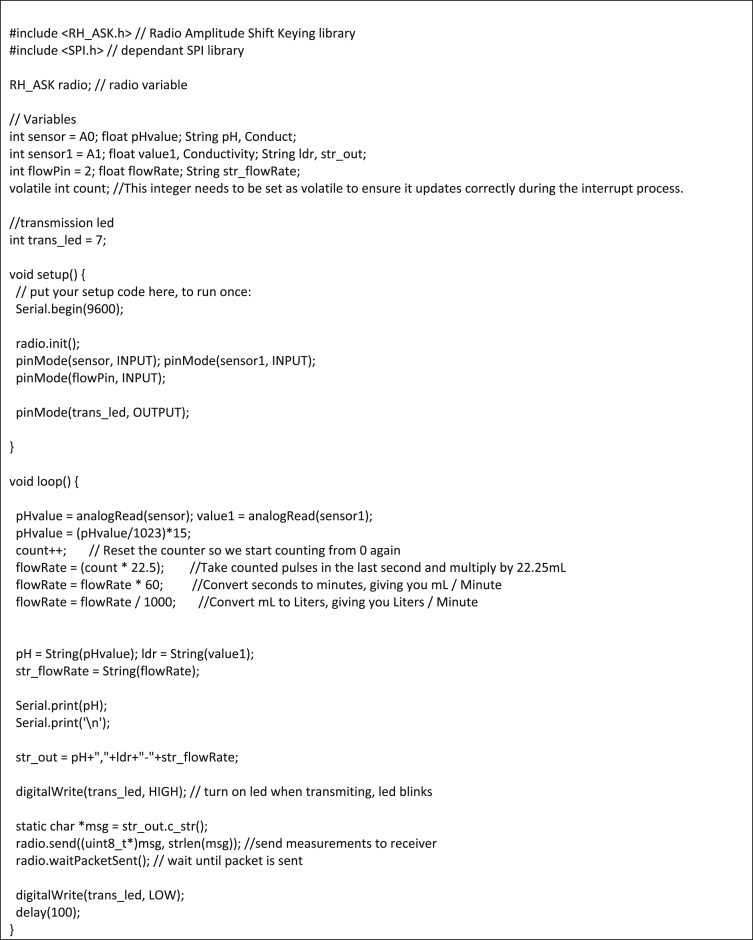

2) Analysis and notification subsystem

**Figure:**