# Supplementary figures and images for: Dataset for a wireless sensor network based drinking-water quality monitoring and notification system
Source: Data Brief. 2019 Nov 16;27:104813. doi: 10.1016/j.dib.2019.104813 (PMC6879973; doi:10.1016/j.dib.2019.104813)

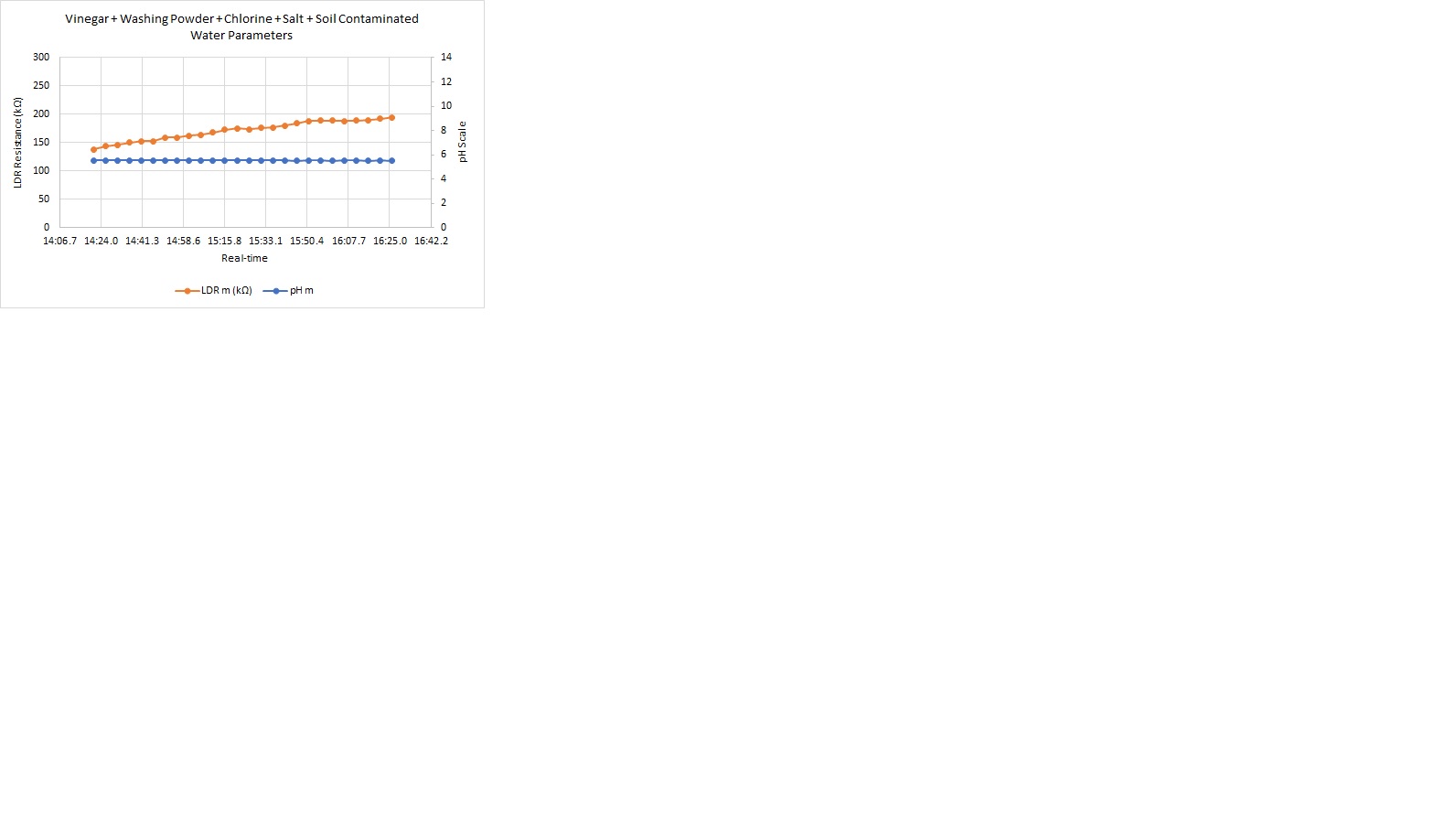

Supplement: Multimedia component 1 [file mmc1.zip › Raw Dataset_DIB/Fig. 10_Vinegar+WashingPowder+Chlorine+Salt+Soil.jpg]

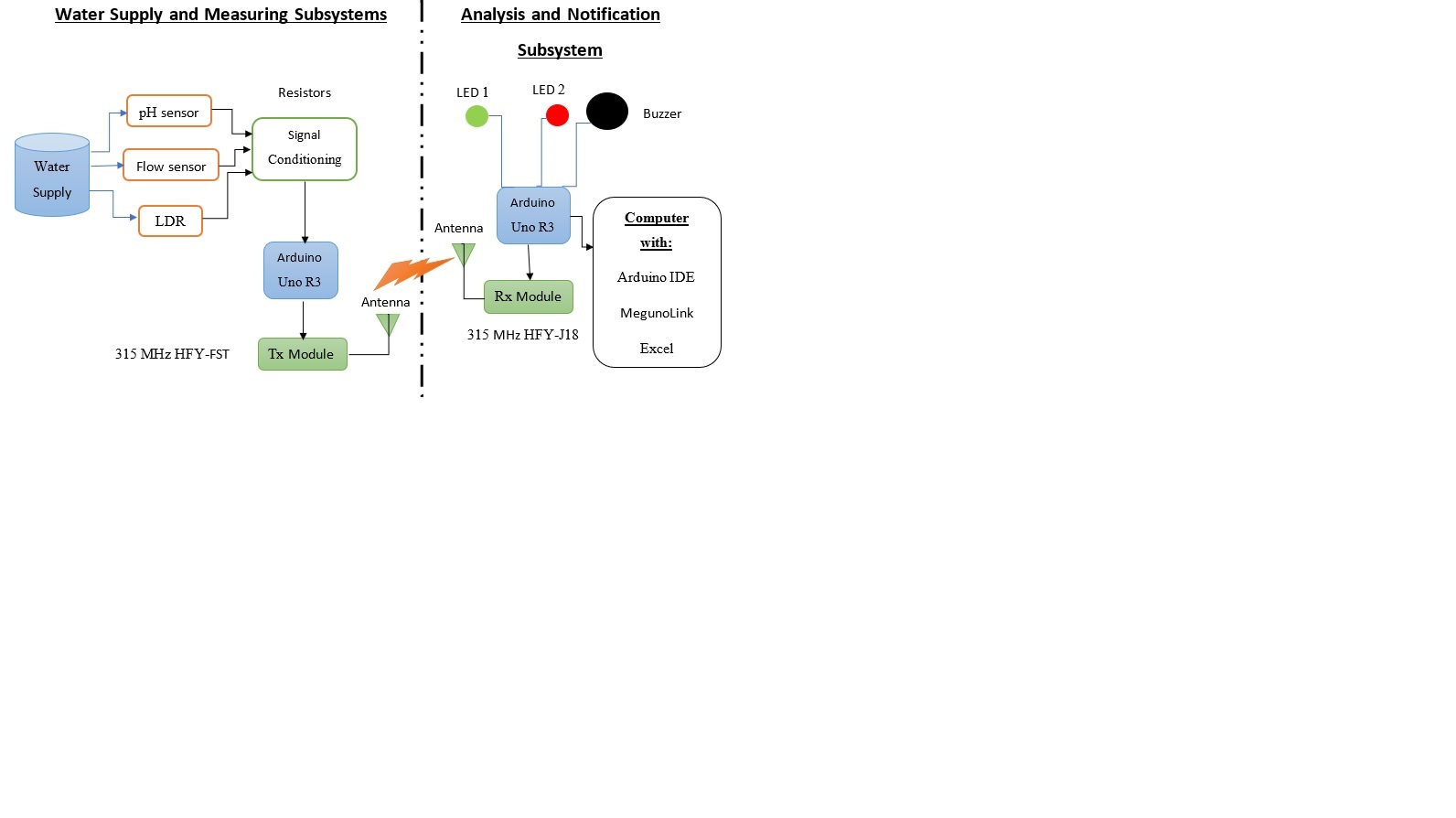

Supplement: Multimedia component 1 [file mmc1.zip › Raw Dataset_DIB/Fig. 11_Block Diagram Water Supply System.jpg]

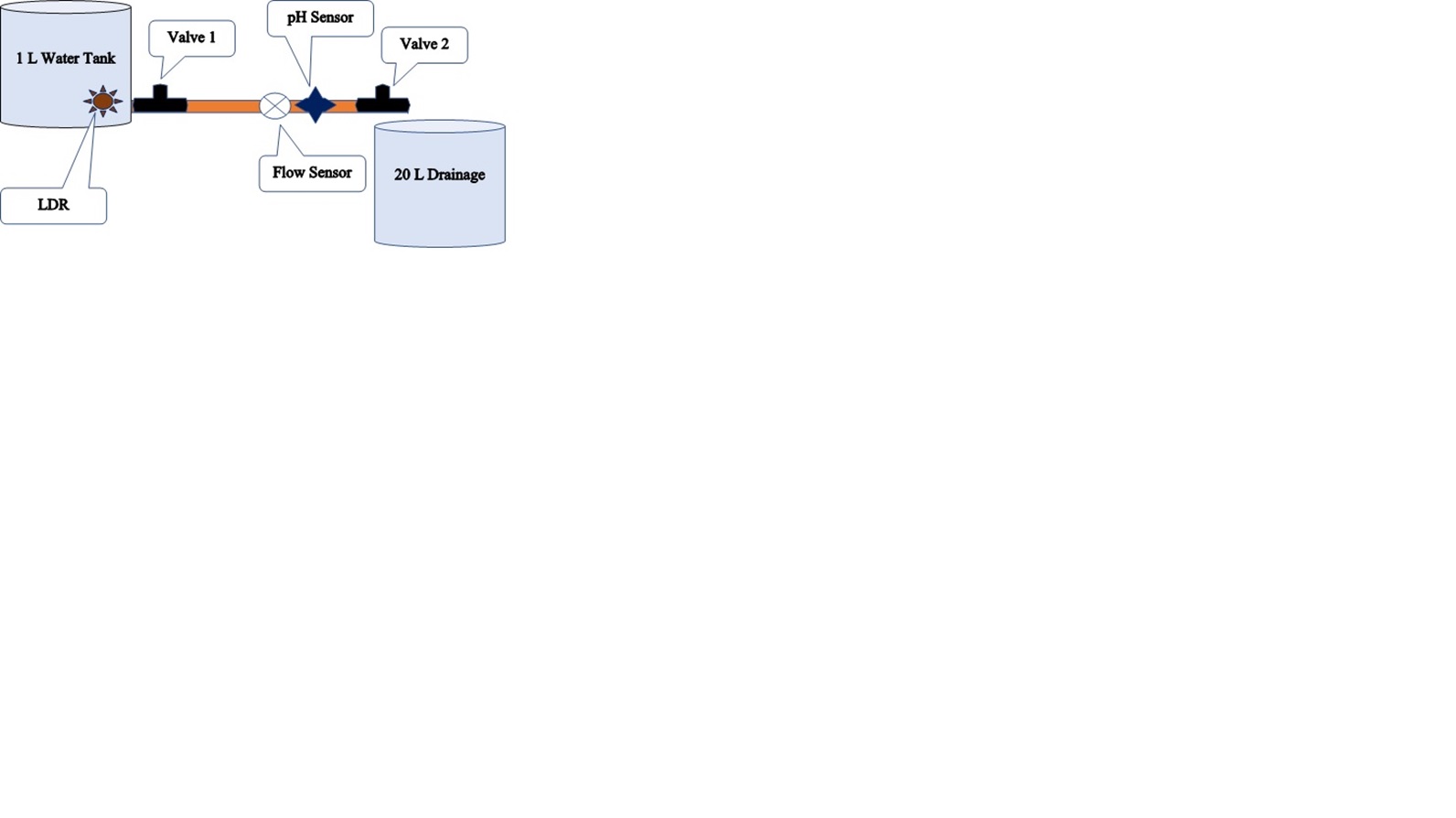

Supplement: Multimedia component 1 [file mmc1.zip › Raw Dataset_DIB/Fig. 12_Schematic Diagram of Water Supply System.jpg]

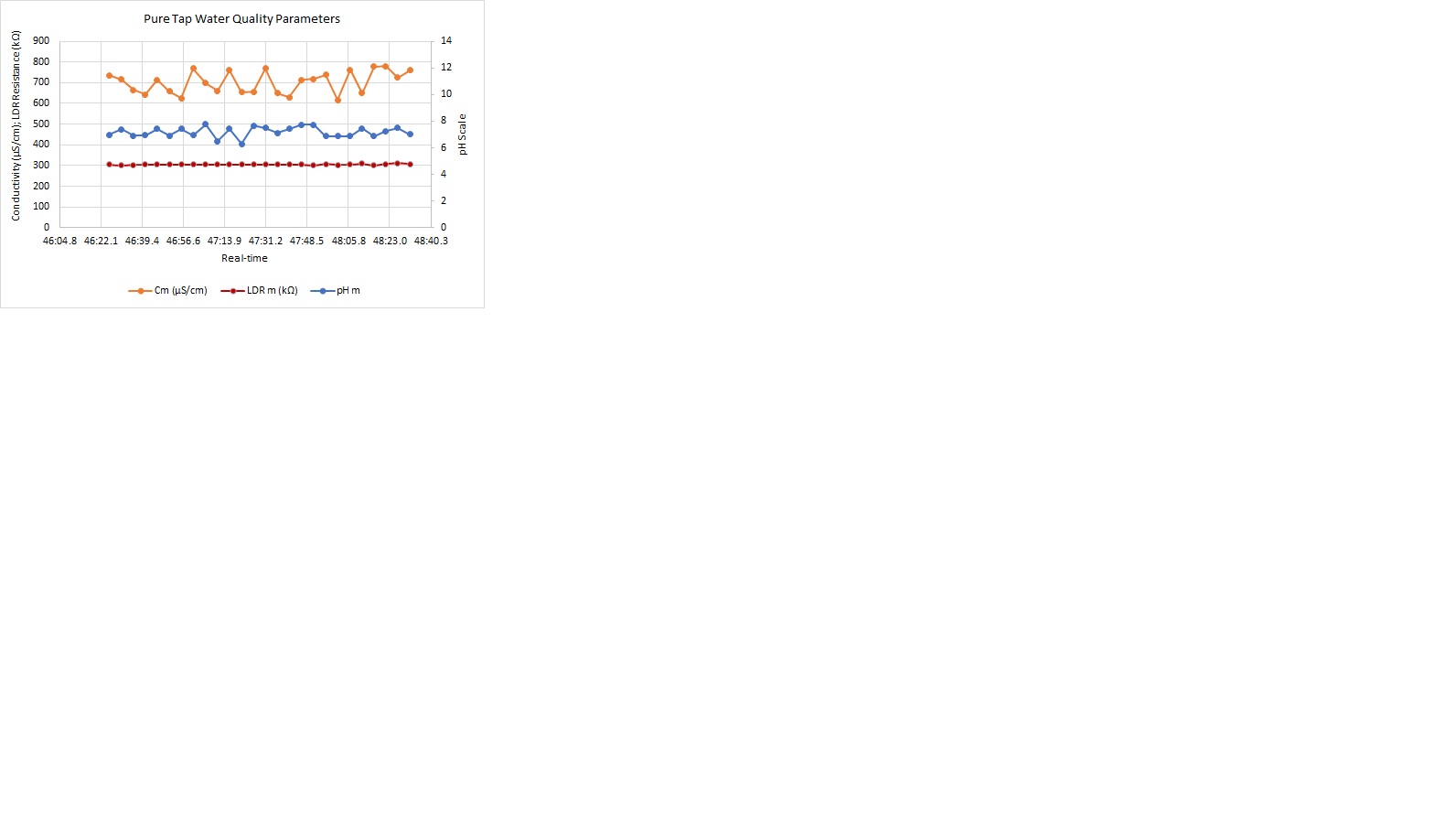

Supplement: Multimedia component 1 [file mmc1.zip › Raw Dataset_DIB/Fig. 1_Pure tap water.jpg]

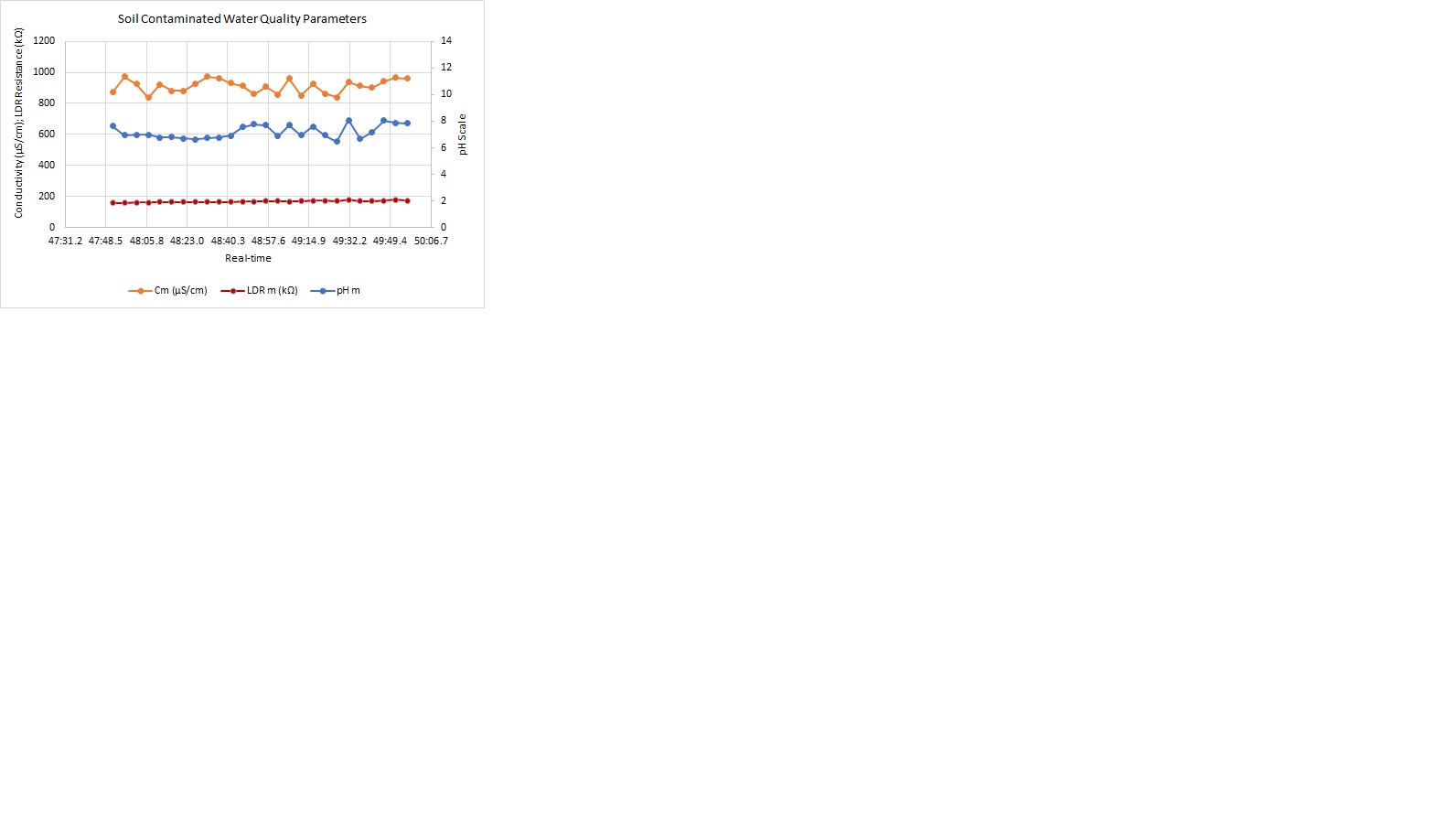

Supplement: Multimedia component 1 [file mmc1.zip › Raw Dataset_DIB/Fig. 2_Soil.jpg]

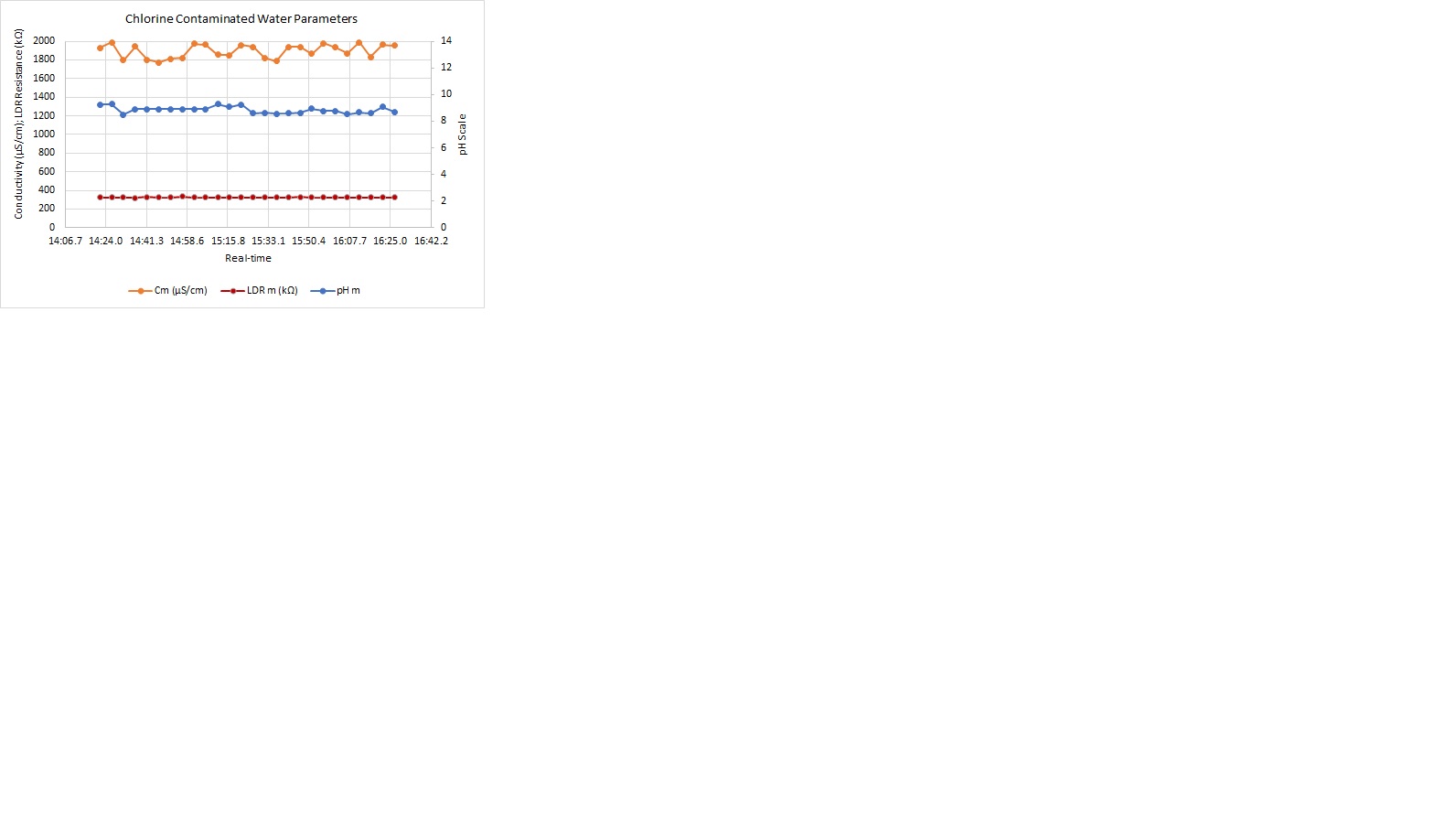

Supplement: Multimedia component 1 [file mmc1.zip › Raw Dataset_DIB/Fig. 3_Chlorine.jpg]

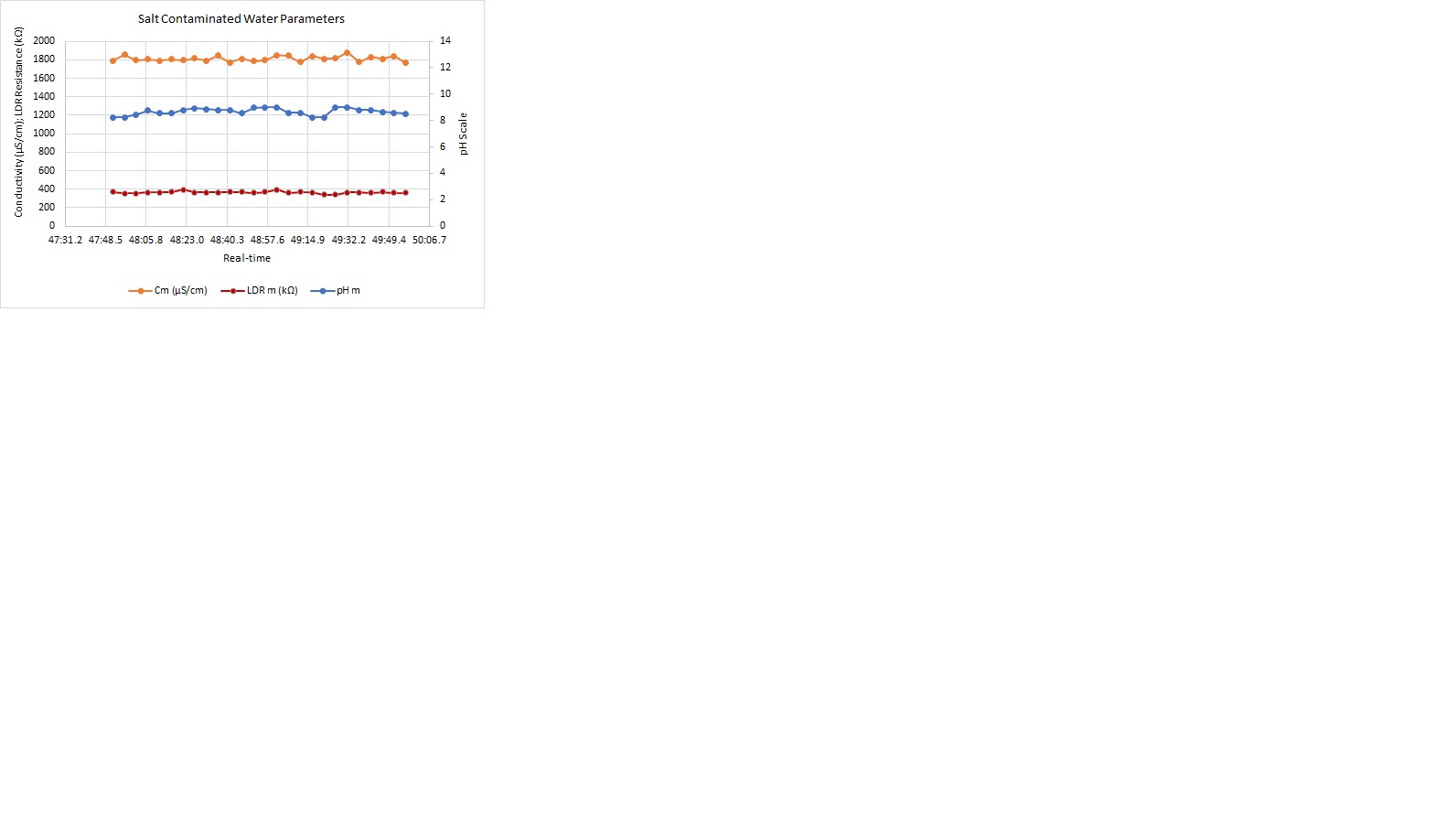

Supplement: Multimedia component 1 [file mmc1.zip › Raw Dataset_DIB/Fig. 4_Salt.jpg]

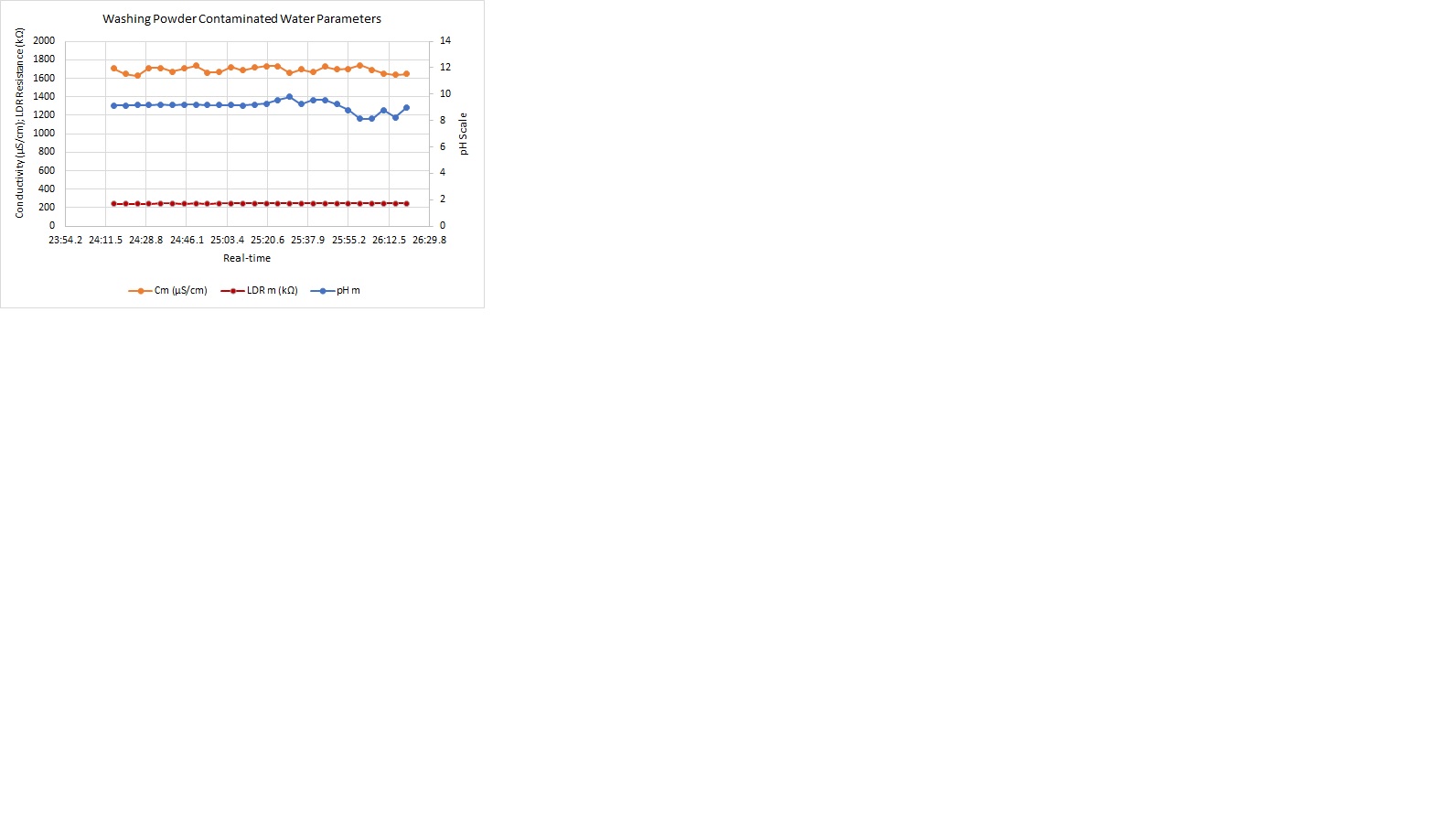

Supplement: Multimedia component 1 [file mmc1.zip › Raw Dataset_DIB/Fig. 5_washingPowder.jpg]

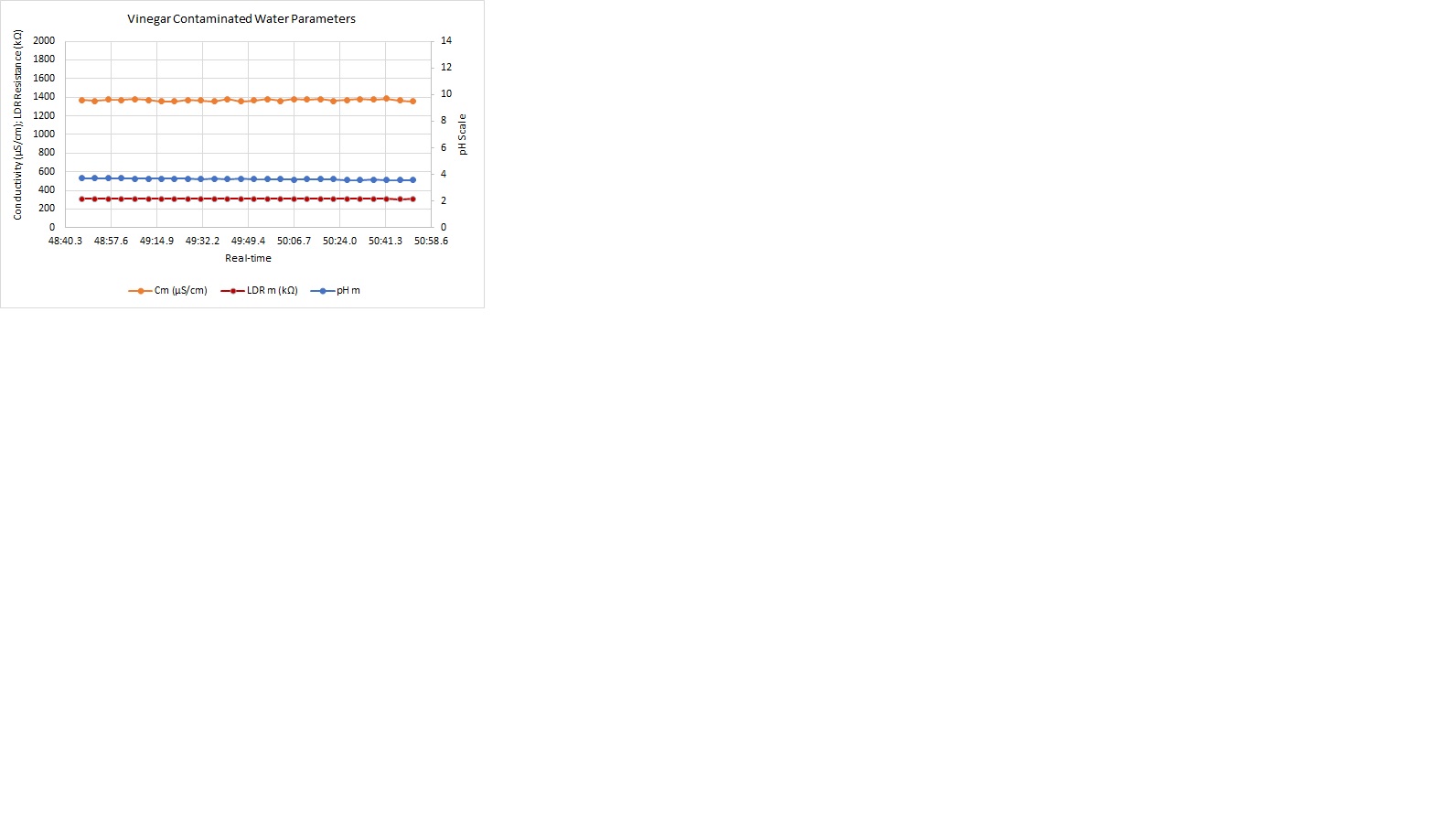

Supplement: Multimedia component 1 [file mmc1.zip › Raw Dataset_DIB/Fig. 6_Vinegar.jpg]

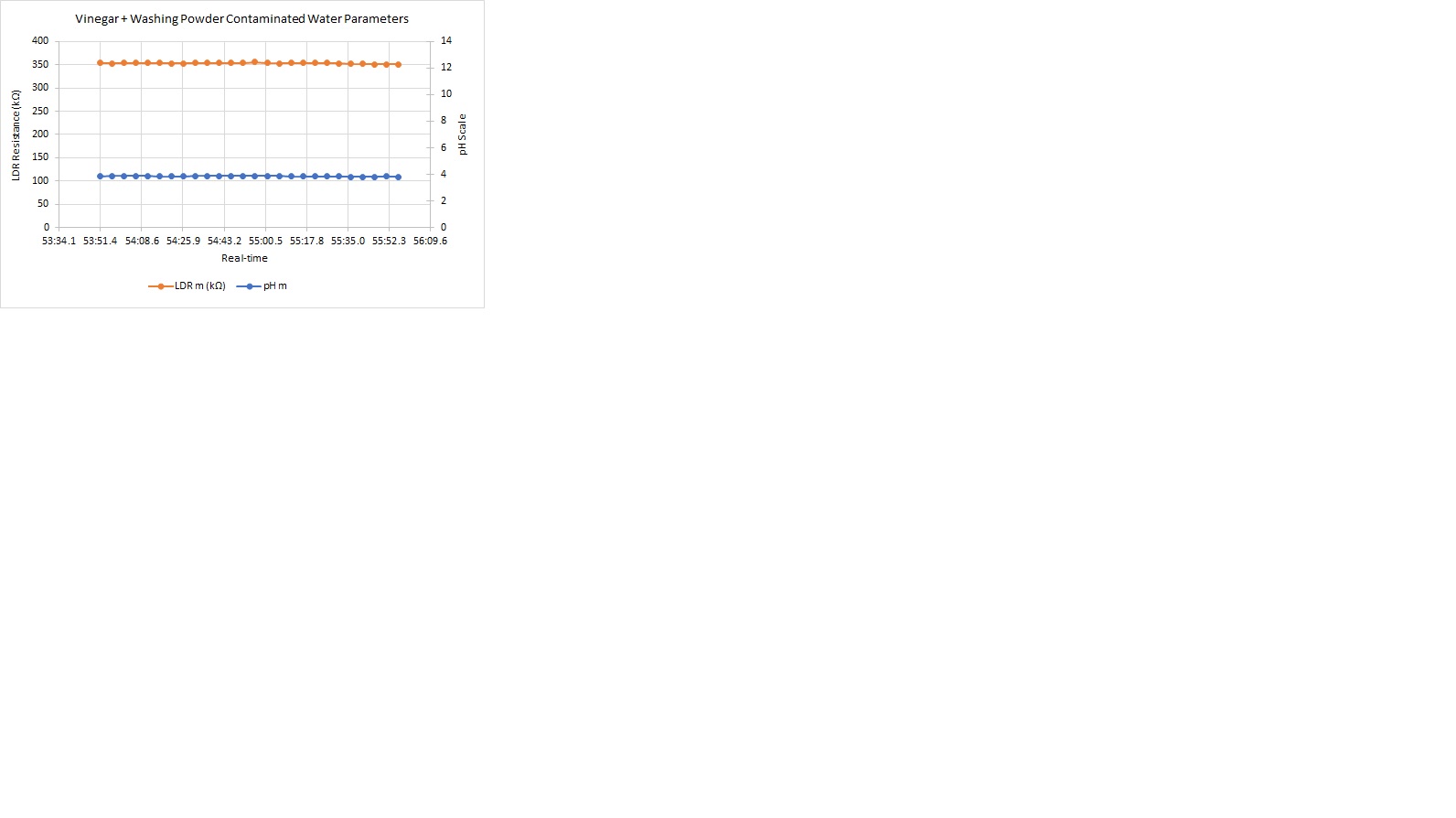

Supplement: Multimedia component 1 [file mmc1.zip › Raw Dataset_DIB/Fig. 7_Vinegar+WashingPowder.jpg]

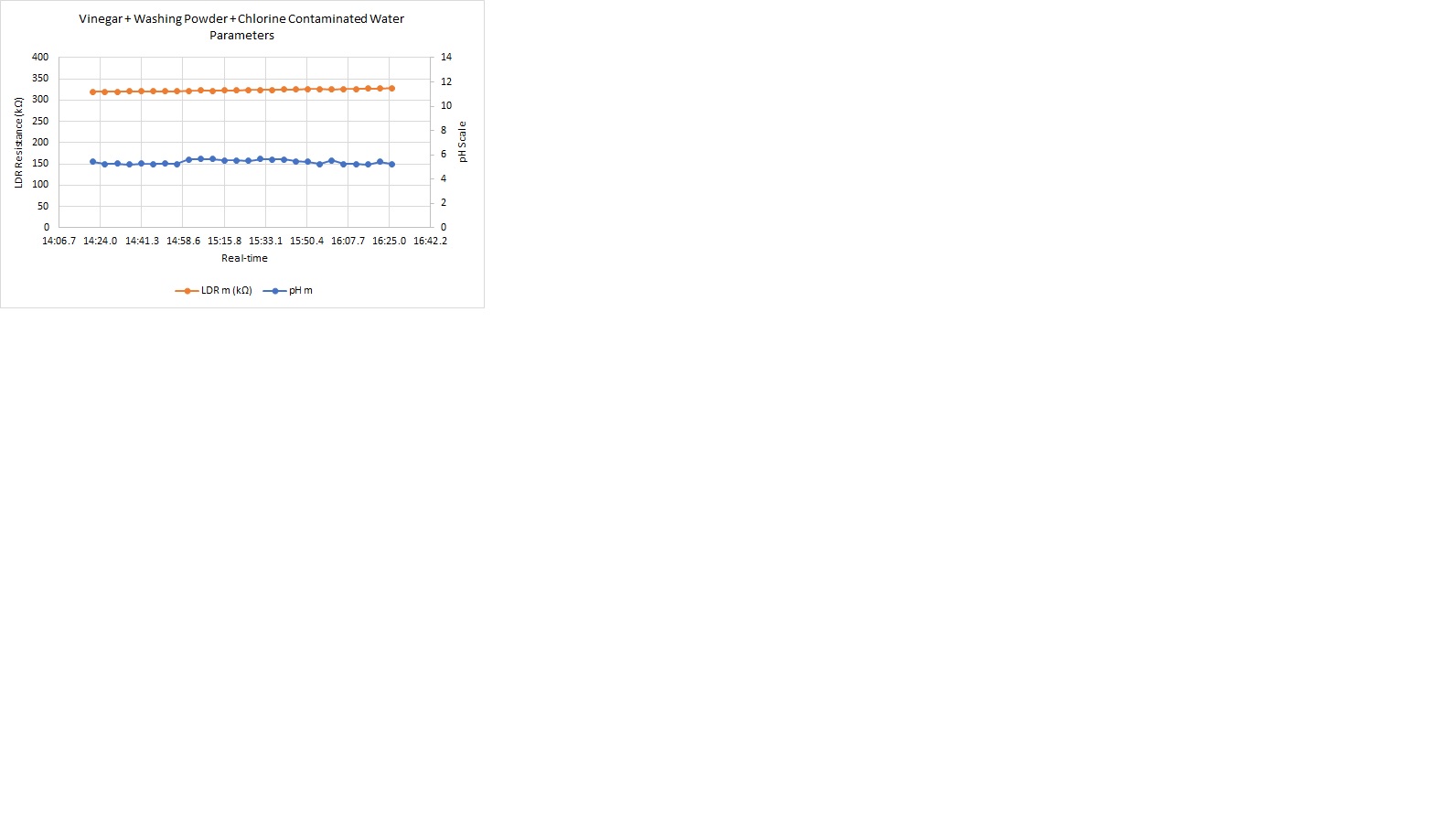

Supplement: Multimedia component 1 [file mmc1.zip › Raw Dataset_DIB/Fig. 8_Vinegar+WashingPowder+Chlorine.jpg]

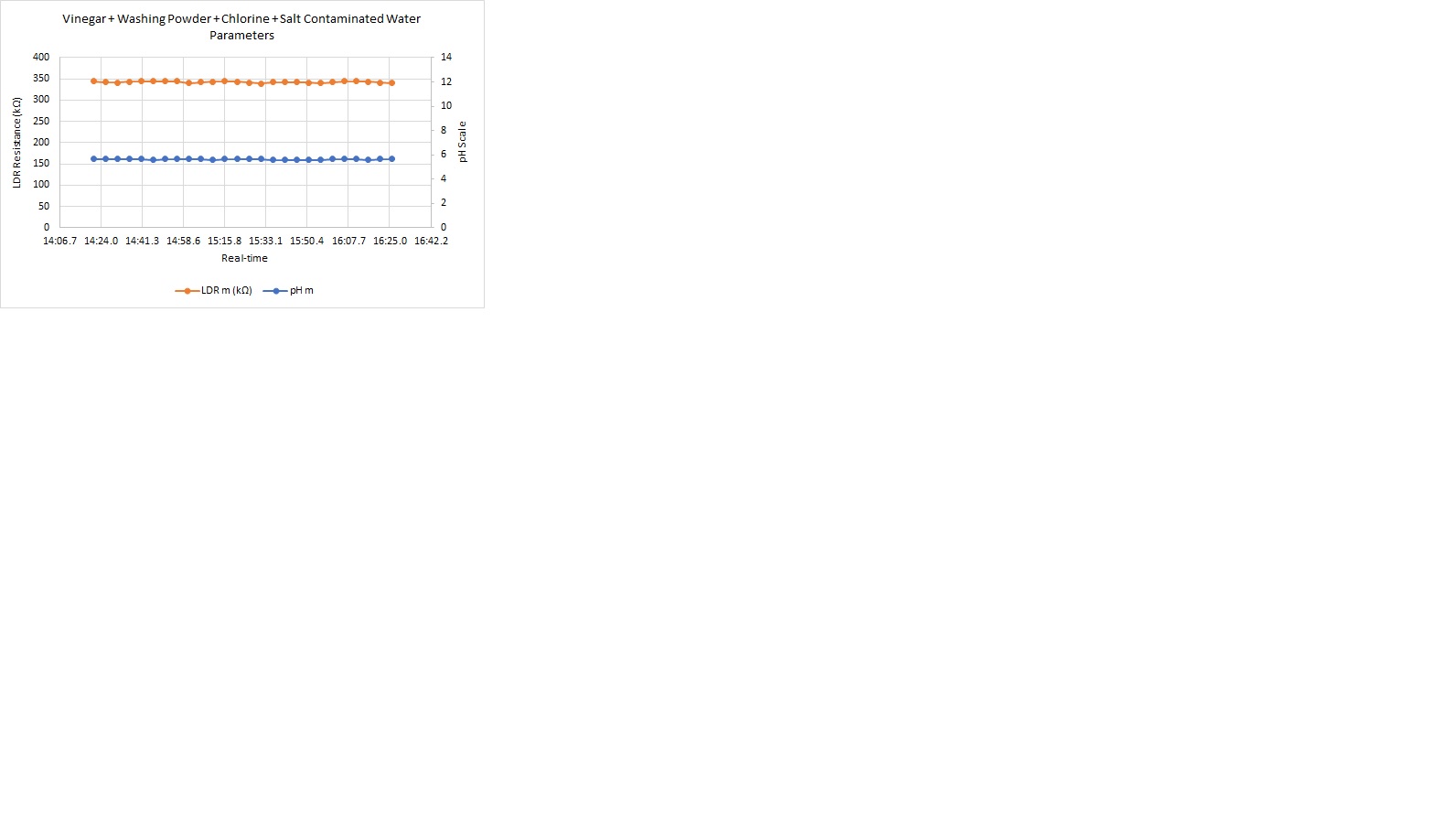

Supplement: Multimedia component 1 [file mmc1.zip › Raw Dataset_DIB/Fig. 9_Vinegar+WashingPowder+Chlorine+salt.jpg]
